# Aberrant neurovascular coupling in Leber’s hereditary optic neuropathy: Evidence from a multi-model MRI analysis

**DOI:** 10.3389/fnins.2022.1050772

**Published:** 2023-01-10

**Authors:** Yi Ji, Ling Wang, Hao Ding, Qin Tian, Ke Fan, Dapeng Shi, Chunshui Yu, Wen Qin

**Affiliations:** ^1^Tianjin Key Lab of Functional Imaging, Department of Radiology, Tianjin Medical University General Hospital, Tianjin, China; ^2^Department of Medical Imaging, Henan Provincial People’s Hospital, Zhengzhou, China; ^3^School of Medical Imaging, Tianjin Medical University, Tianjin, China; ^4^Henan Eye Institute, Henan Eye Hospital, Henan Provincial People’s Hospital, Zhengzhou University People’s Hospital, Zhengzhou, China

**Keywords:** Leber’s hereditary optic neuropathy, mitochondrial disease, arterial spin labeling, regional homogeneity, neurovascular coupling, cerebral blood flow, functional magnetic resonance imaging

## Abstract

The study aimed to investigate the neurovascular coupling abnormalities in Leber’s hereditary optic neuropathy (LHON) and their associations with clinical manifestations. Twenty qualified acute Leber’s hereditary optic neuropathy (A-LHON, disease duration ≤ 1 year), 29 chronic Leber’s hereditary optic neuropathy (C-LHON, disease duration > 1 year), as well as 37 healthy controls (HCs) were recruited. The neurovascular coupling strength was quantified as the ratio between regional homogeneity (ReHo), which represents intrinsic neuronal activity and relative cerebral blood flow (CBF), representing microcirculatory blood supply. A one-way analysis of variance was used to compare intergroup differences in ReHo/CBF ratio with gender and age as co-variables. Pearson’s Correlation was used to clarify the association between ReHo, CBF, and neurovascular coupling strength. Furthermore, we applied linear and exponential non-linear regression models to explore the associations among ReHo/CBF, disease duration, and neuro-ophthalmological metrics. Compared with HCs, A_LHON, and C_LHON patients demonstrated a higher ReHo/CBF ratio than the HCs in the bilateral primary visual cortex (B_CAL), which was accompanied by reduced CBF while preserved ReHo. Besides, only C_LHON had a higher ReHo/CBF ratio and reduced CBF in the left middle temporal gyrus (L_MTG) and left sensorimotor cortex (L_SMC) than the HCs, which was accompanied by increased ReHo in L_MTG (*p* < 1.85e^–3^, Bonferroni correction). A-LHON and C-LHON showed a negative Pearson correlation between ReHo/CBF ratio and CBF in B_CAL, L_SMC, and L_MTG. Only C_LHON showed a weak positive correlation between ReHo/CBF ratio and ReHo in L_SMC and L_MTG (*p* < 0.05, uncorrected). Finally, disease duration was positively correlated with ReHo/CBF ratio of L_SMC (Exponential: *Radj^2^* = 0.23, *p* = 8.66e^–4^, Bonferroni correction). No statistical correlation was found between ReHo/CBF ratio and neuro-ophthalmological metrics (*p* > 0.05, Bonferroni correction). Brain neurovascular “dyscoupling” within and outside the visual system might be an important neurological mechanism of LHON.

## 1. Introduction

Leber’s hereditary optic neuropathy (LHON) is an inherited genetic disorder caused by mutations in mitochondrial DNA (mtDNA), leading to severe bilateral continuous painless loss of vision, especially in young males (Chinnery et al., 2000). Previous research reported some pathological changes in the anterior visual pathway, such as degeneration in retinal ganglion cells (RGCs), progressive thinning of the retinal nerve fiber layer (RNFL) thickness, the axonal loss of the optic nerve as well as the loss of nerve fibers in the central part (Savini et al., 2005; Balducci et al., 2016; Wang et al., 2017; Asanad et al., 2019). Besides, a case report identified diffuse histopathological brain white matter changes in LHON mimicking gliomatosis cerebri (Saruta et al., 2021). Recent studies have reported widespread brain involvement using advanced neuroimaging techniques such as high-resolution structural magnetic resonance imaging (sMRI), diffusion tensor imaging (DTI), and functional magnetic resonance imaging (fMRI). For example, white matter integrity impairment was found both within (Barcella et al., 2010; Milesi et al., 2012; Rizzo et al., 2012; Manners et al., 2015; Wang et al., 2017; Jonak et al., 2020a) and outside the visual pathways (Wang et al., 2021) in LHON patients and even in asymptomatic carriers (Long et al., 2019). Similarly, reduced gray matter volume (GMV) in the primary visual cortex (Barcella et al., 2010; Tian et al., 2022), thickening of extrastriate cortex thickness (d’Almeida et al., 2013; Mateus et al., 2016), an enlarged ventricular system (Jonak et al., 2020b), and changes in the hippocampus sub-fields volume (Grochowski et al., 2020) were also identified in LHON. In addition, some researchers also found decreased spontaneous neural activity in the associated visual areas (Rocca et al., 2011; d’Almeida et al., 2013; Jonak, 2020), and brain regions outside the visual cortex both showed increased (Rocca et al., 2011; Tian et al., 2022) or decreased brain activity (Jonak, 2020). These results suggested that the brain’s impairment was not confined to the visual system.

The LHON mtDNA mutation occurs in the gene encoding mitochondrial complex I of the electron transport chain, also known as the nicotinamide adenine dinucleotide dehydrogenase subunit (ND1). ND1 produces adenosine triphosphates (ATPs) under aerobic conditions (Hirst, 2013). LHON mutation changes in a single amino acid of ND1 that exhausts the energy in neuron cells and in turn causes the death of neurons (Kirches, 2011). In a previous study, all three types of LHON mutation-carrying cell cultures detected a rapid decrease in ATP concentration (Kogachi et al., 2019). In addition to the decrease in ATPs’ production, the damage to the glutamate transport system and elevating oxidative stress also lead to RGC loss in LHON (Zhuo et al., 2012). The activity of brain tissue relies on the aerobic oxidation of oxygen and glucose for energy. It is well known that increased neuronal activity is accompanied by increased regional metabolic rate and cerebral blood flow (CBF), indicating the close coupling between neuronal activity and microcirculatory blood supply (termed neurovascular coupling) (Iadecola et al., 1993; Chaigneau et al., 2003). The blood-brain barrier separates blood from brain tissue, but blood vessel cells, neighboring neurons, and astrocytes can still communicate interactively *via* the neurovascular unit (NVU) (Iadecola, 2017), which plays a bridge role in information transmission (Harder et al., 2002; Lopez-Bayghen and Ortega, 2011; Santello et al., 2012). By synthesizing and releasing vasoactive substances, NVU can effectively dilate or contract blood vessels and cause CBF changes (Zonta et al., 2003). Previous studies have shown that multi-modal neuroimages comprising both regional CBF and fMRI can provide a more comprehensive picture of neurovascular coupling abnormalities in patients, such as end-stage renal disease (Jin et al., 2020), neuromyelitis optica (Guo et al., 2019), type 2 diabetes mellitus (Hu et al., 2019a; Yu et al., 2019, Zhang et al., 2021b), chronic migraine (Hu et al., 2019b), and schizophrenia (Zhu et al., 2017). As mentioned above, in LHON patients, early studies have reported abnormal spontaneous neural activity (Rocca et al., 2011; Vacchiano et al., 2019; Jonak, 2020) and mtDNA-induced energy reduction in RGC (Zhang et al., 2021a) from a unimodal perspective. However, to our knowledge, no studies have attempted to investigate the relationship between abnormal brain activity and brain metabolism in LHON. Elucidating this issue would deepen our understanding of the neurological mechanisms of LHON-related brain injury and provide a potential basis for early clinical intervention.

This study aims to elucidate whether the neurovascular coupling was disrupted in both acute and late LHON. The neurovascular coupling index was quantified as the ratio between regional homogeneity (ReHo) and relative CBF (Li et al., 2012; Guo et al., 2019). ReHo is a resting-state fMRI (rfMRI) measure that quantifies the consistency of the time series between a single voxel and its adjacent voxels (Zang et al., 2004), which is frequently applied to represent regional spontaneous neuronal activity. CBF derived from a non-invasive arterial spin labeling (ASL) technique is a microcirculatory blood supply measure that quantifies the blood flow change in unit brain tissue within a certain period (Detre et al., 2012; Alsop et al., 2015). Early studies have shown differences in structural abnormality between the acute and chronic LHON (Wang et al., 2021; Zhang et al., 2021a). Thus, we split the LHON patients into acute and chronic sub-groups and applied voxel-based statistics to explore the possible ReHo/CBF ratio abnormality in each LHON sub-group and the difference between them. Furthermore, we studied the corresponding changes in CBF and ReHo in brain regions with abnormal ReHo/CBF ratios. Finally, linear and non-linear regressions were used to investigate the potential relationship among ReHo/CBF ratio, disease duration, and neuro-ophthalmological metrics.

## 2. Materials and methods

### 2.1. Participants

We initially recruited 55 LHON patients diagnosed in Zhengzhou University People’s Hospital from May 2012 to December 2016. These participants have also been involved in several previous studies (Long et al., 2019; Wang et al., 2021; Zhang et al., 2021a; Tian et al., 2022). Briefly, the inclusion criteria were: (1) carrying LHON mtDNA mutations; (2) no history of other ophthalmic, neurological, psychiatric, major medical conditions, or substance abuse; (3) no visible brain lesions (Supplementary Figures 1, 2); (4) no MRI contraindications. All patients had idebenone treatment during the acute phase for 1 week to 3 months before MRI examination. The LHON patients covered a wide range of disease duration spanning the acute and chronic phases (from 3 weeks to 422 months). Thus, we further separated them into 23 acute Leber’s hereditary optic neuropathy (A-LHON, disease duration ≤ 1 year) and 32 chronic Leber’s hereditary optic neuropathy (C-LHON, disease duration > 1 year) patients based on the duration (Wang et al., 2021). It should be noted some of the patients overlapped with early studies by our team (Wang et al., 2017; Wang et al., 2021; Zhang et al., 2021a; Tian et al., 2022), but the contents of this study were independent of these early works.

Three A-LHON were excluded due to no neuro-ophthalmological examination (1 case) and poor CBF image quality (2 cases). Three C-LHON were excluded due to a missing T1 image for normalization (1 case), poor CBF image quality (1 case), and severe head motion on rfMRI (1 case). Thus, this study finally enrolled 20 qualified A-LHON (ages ranging from 10 years to 57 years old, 18 males, 14 cases of m.11778G > A, 1 case of m.3460G > A, and 5 cases of m.14484T > C) and 29 C-LHON (ages from 13 years to 53 years old, 19 males, 23 cases of m.11778G > A, 2 cases of m.3460G > A, and 4 cases of m.14484T > C). We also recruited 37 gender and age coarsely matched healthy controls (HCs, ages 11 years to 44 years old, 27 males) with the same enrollment criteria except for no visual impairment and mtDNA mutations.

The research was approved by the Ethics Committees of Henan Provincial People’s Hospital and was carried out in compliance with the Code of Ethics of the World Medical Association (Declaration of Helsinki). Written informed consent was obtained from all subjects or their legal guardians.

### 2.2. Neuro-ophthalmological examination

The logarithm of the minimum angle of resolution (logMAR) was used to evaluate the corrected visual acuity. Octopus perimeter 101G2 program TsOP Strategy (Interzeag AG, Haig-Streit Schlieren, Switzerland) was used to examine the visual field represented by the mean defect (MD), mean sensitivity (MS), and loss of variance (LV). The peripapillary retinal nerve fiber layer (RNFL) thickness was measured by optical coherence tomography (Carl Zeiss Meditec, Dublin, CA, USA) with a preset diameter of 3.45 mm.

### 2.3. MRI data acquisitions

MRI data were obtained by a 3.0T MR scanner (Discovery MR750, GE Healthcare, Waukesha, WI, USA). Resting-state perfusion data were acquired by a pseudo-continuous ASL (pcASL) sequence with a 3D fast spin-echo acquisition and background suppression. The ASL scanning parameters included: repetition time (TR) = 4632 ms; echo time (TE) = 10.5 ms; post-labeling delay (PLD) = 1525 ms; flip angle (FA) = 111°; field of view (FOV) = 230 mm × 230 mm; matrix = 128 × 128; slice thickness = 4 mm; 36 continuous slices; resulting in a voxel size of 1.8 mm × 1.8 mm × 4 mm. High-resolution three-dimensional T1-weighed images (T1WI) were obtained using a fast-spoiled gradient echo sequence with the following parameters: TR = 8.2 ms; TE = 3.2 ms; inversion time (TI) = 450 ms; FA = 12°; FOV = 256 mm × 256 mm; matrix = 256 × 256; slice thickness = 1 mm; 176 continuous slices; voxel size = 1 mm × 1 mm × 1 mm. RfMRI data were acquired using a gradient-echo echo-planar imaging sequence with the following parameters: TR = 2000 ms; TE = 30 ms; FA = 90°; FOV = 240 mm × 240 mm; matrix = 64 × 64; No. time points = 210; slice thickness = 4 mm; 33 slices; no gap; voxel size = 3.75 mm × 3.75 mm × 4 mm.

### 2.4. Data preprocessing and neurovascular coupling quantification

The ASL images of each subject were coregistered to his/her T1 images after skull stripping. Then T1 images of each subject were segmented and normalized into Montreal Neurological Institute (MNI) space based on the algorithm named diffeomorphic anatomical registration through the exponentiated Lie algebra (DARTEL). Next, the CBF map (automatically generated during the scan) was transformed into the MNI space using the DARTEL parameters and was resliced into a voxel size of 2 mm × 2 mm × 2 mm. After that, the normalized CBF images were skull-stripped and scaled by the global mean CBF value of the brain. Finally, the scaled CBF map was spatially smoothed with a Gaussian kernel of 6 mm × 6 mm × 6 mm full-width at half maximum (FWHM).

The first 10 time points were discarded to allow for magnetization equilibrium. Then the remaining 200 volumes of rfMRI images were undergone slice time correction, motion correction, spatial normalization (like ASL based on DARTEL), nuisance covariate regression [including the linear trend, the average signals of the white matter, cerebrospinal fluid, motion parameters based on the Friston-24 model, and spike volume with framewise displacement exceed 0.5 mm (Power et al., 2012)], and band-pass filtering (0.01–0.10 Hz). The ReHo map was calculated using the preprocessed rfMRI data by the Kendall harmony coefficient (KCC) of a voxel with its 26 neighbors and was scaled by his/her brain’s global mean (Zang et al., 2004). Finally, the ReHo map was smoothed with an isotropic Gaussian kernel of 6 mm × 6 mm × 6 mm FWHM.

We calculated the neurovascular coupling index as the ratio between each subject’s preprocessed ReHo map and his/her CBF map voxel-by-voxel (Li et al., 2012; Guo et al., 2019). These steps were carried out using a self-coded pipeline developed based on SPM12^1^ and DPABI V2.3.^2^

### 2.5. Statistical analysis

A voxel-based one-way ANOVA was used to compare ReHo/CBF ratio differences between the three groups with age and gender as covariates [voxel-wise *p* < 1.00e^–3^, cluster-wise family wise error (FWE) corrected *p* < 0.05 (cluster size > 1147 voxels)]. Then we extracted the average ReHo/CBF ratio, ReHo value, and CBF value of the region of interest (ROI) with voxels that survived in voxel-wise ANOVA and performed *post hoc* analyses (*p* < 0.05/3 ROIs/3 measures/3 comparison pairs = 1.85e^–3^, Bonferroni corrected). Moreover, we conducted Pearson’s Correlation to clarify if the strengths of ReHo or CBF were associated with the neurovascular coupling strength (*p* < 0.05/3 ROIs/2 groups/2 metric pairs = 4.17e^–3^, Bonferroni corrected).

A Chi-square test was used to inter-group sex differences. In addition, one-way ANOVA (among three groups) or two-sample *t*-tests (between two groups) were used to compare the intergroup differences in continuous variables, including age, disease duration, and neuro-ophthalmological metrics (*p* < 0.05).

To investigate the potential association between neurovascular coupling index and clinical measures, we applied several linear and non-linear exponential regressions analyses between ReHo/CBF ratio and clinical measures such as disease duration and neuro-ophthalmological measures, respectively, after regressing covariates of gender and age (*p* < 0.05/3 ROIs/5 measures/2 models = 1.67e^–3^, Bonferroni correction).

The voxel-wise statistic was conducted using SPM12.^3^ All table data (demographics, clinical measurements, and ROI-wise data) underwent statistics by SPSS19.0.^4^

## 3. Results

### 3.1. Demographic data and clinical variables

The demographic and clinical characteristics are summarized in Table 1. No intergroup differences were found in age (*F* = 1.86, *p* = 1.62e^–1^) and gender (Chi-square test, χ^2^ = 4.72, *p* = 9.51e^–2^). ANOVA revealed significant differences in MD (*F* = 48.76, *p* = 3.11e^–14^), MS (*F* = 54.39, *p* = 4.74e^–15^), LV (*F* = 21.74, *p* = 4.12e^–8^), and peripapillary RNFL thickness (*F* = 72.55, *p* = 4.46e^–18^) among the A-LHON, C-LHON, and HCs. *Post hoc* test demonstrated A-LHON had higher MD (*p* = 1.10e^–7^), higher LV (*p* = 1.03e^–3^) and lower MS (*p* = 1.84e^–7^) than HCs. C-LHON patients had higher MD (*p* = 8.80e^–15^), higher LV (*p* = 7.43e^–9^), lower MS (*p* = 1.13e^–15^), and thinner RNFL thickness (*p* = 3.87e^–16^) than HCs. No significant difference was found in the mtDNA mutation distribution between the acute and chronic LHON (Fisher’s exact test, *p* = 6.77e^–1^).

**TABLE 1:** Demographic and clinical characteristics of this study.

	Acute LHON	Chronic LHON	HCs	Total effects		A-LHON vs. HCs	C-LHON vs. HCs	A-LHON vs. C-LHON
				*F/T/χ^2^*	*p*	*p*	*p*	*p*
Age (years)	21.55 ± 11.28	27.76 ± 12.04	24.96 ± 10.16	*F* = 1.86	*p* = 1.62e^–1^	–	–	–
Gender (male/female)	18/2	18/11	27/10	χ^2^ = 4.72	*p* = 9.51e^–2^	–	–	–
Duration (months)	4.72 ± 4.01	121.79 ± 129.04	–	*t* = -4.04	–	–	–	–
MD (dB)	13.35 ± 9.27	18.10 ± 7.99	1.49 ± 1.11	*F* = 48.76	*p* = 3.11e^–14^*	1.10e^–7^*	8.80e^–15^*	2.13e^–2^*
MS (dB)	15.83 ± 8.07	10.92 ± 8.15	27.60 ± 1.18	*F* = 54.39	*p* = 4.74e^–15^*	1.84e^–7^*	1.13e^–15^*	1.91e^–2^*
LV (dB^2^)	24.91 ± 25.29	37.14 ± 25.27	4.045 ± 1.97	*F* = 21.74	*p* = 4.12e^–8^*	1.03e^–3^*	7.43e^–9^*	5.23e^–2^
RNFL thickness (μm)	104.53 ± 25.21	60.20 ± 11.08	100.23 ± 6.99	*F* = 72.55	*p* = 4.46e^–18^*	3.29e^–2^*	3.87e^–16^*	1.98e^–15^*
mtDNA.11778G > A	14	23	–	*p* = 6.77e^–1#^		–	–	–
mtDNA.14484T > C	5	4	–			–	–	–
mtDNA.3460G > A	1	2	–			–	–	–

Data were reported as mean ± SD, and significant differences were labeled with asterisks (*). ^#^Fisher exact test. LHON, Leber’s hereditary optic neuropathy; A-LHON, acute Leber’s hereditary optic neuropathy; C-LHON, chronic Leber’s hereditary optic neuropathy; HCs, healthy controls; MD, mean defect; MS, mean sensitivity; LV, loss of variance; RNFL, retinal nerve fiber layer.

### 3.2. ReHo/CBF ratio changes in LHON patients

**FIGURE 1:**
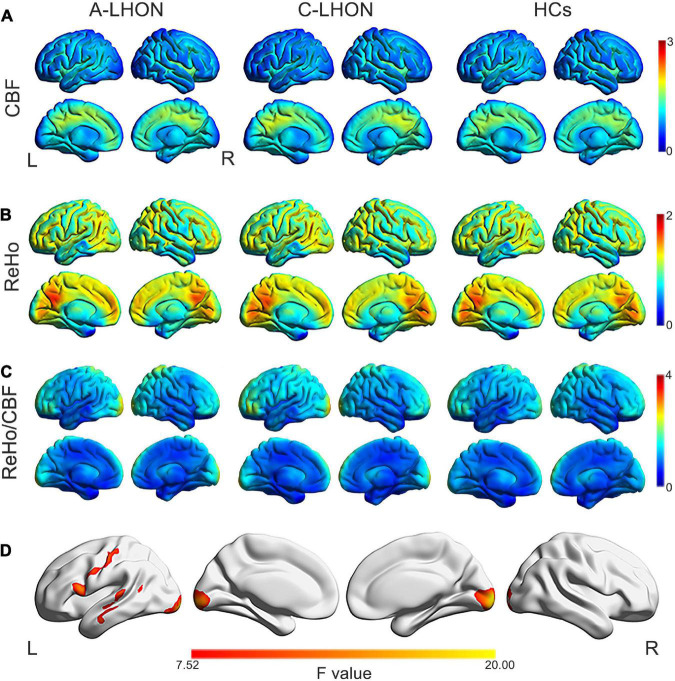
ReHo, CBF, and ReHo/CBF ratio maps and intergroup differences in ReHo/CBF ratio. Average CBF **(A)**, ReHo **(B)**, and ReHo/CBF ratio **(C)** maps are present for A-LHON (left), C-LHON patients (middle), and HCs (right). **(D)** Represents one-way analysis of variance for intergroup differences in ReHo/CBF ratio between the three groups (*p* < 1.00e^– 3^, family wise error corrected at the cluster level). CBF, cerebral blood flow; ReHo, regional homogeneity; A-LHON, acute Leber’s hereditary optic neuropathy; C-LHON, chronic Leber’s hereditary optic neuropathy; HCs, healthy controls.

**FIGURE 2:**
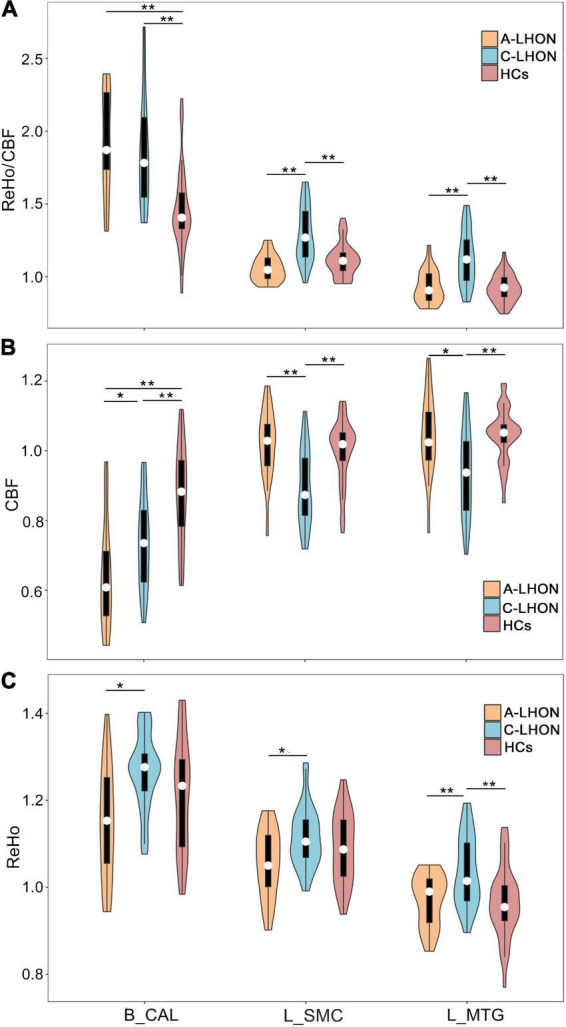
Differences in ReHo/CBF ratio, CBF, and ReHo values between each pair of groups. *Survival under a nominal *p* < 0.05, **survival under a Bonferroni-corrected *p* < 0.05 (equal normal *p* < 1.85e^– 3^). CBF, cerebral blood flow; ReHo, regional homogeneity; A-LHON, acute Leber’s hereditary optic neuropathy; C-LHON, chronic Leber’s hereditary optic neuropathy; HCs, healthy controls; B_CAL, bilateral calcarine fissure and surrounding cortex; L_MTG, left middle temporal gyrus; L_SMC, left sensorimotor cortex.

The average ReHo, CBF, and their derived ReHo/CBF ratio maps for each group are shown in Figures 1A–C. LHON patients demonstrated abnormal ReHo/CBF ratio in the bilateral calcarine fissure and surrounding cortex (B_CAL), left sensorimotor cortex (L_SMC), and left middle temporal gyrus (L_MTG) (voxel-wise *p* < 1.00e^–3^, FWE corrected cluster size > 1147 voxels) (Figure 1D and Table 2). *Post hoc* analyses identified a higher ReHo/CBF ratio in A_LHON and C_LHON patients than the HCs in the B_CAL. Besides, C_LHON patients had a higher ReHo/CBF ratio in L_SMC and L_MTG than the A_LHON and HCs (*p* < 1.85e^–3^, Bonferroni correction) (Figure 2A).

**TABLE 2:** Brain regions showing altered ReHo/CBF ratio among A-LHON, C-LHON, and HCs.

Brain regions	Cluster voxel size	Peak *F*-value	Peak MNI coordinates (mm)		
			*x*	*y*	*z*
B_CAL	2524	20.78	10	–100	0
L_SMC	1517	16.40	–68	–34	2
L_MTG	1147	14.72	–58	12	26

B_CAL, bilateral calcarine fissure and surrounding cortex; L_MTG, left middle temporal gyrus; L_SMC, left sensorimotor cortex; MNI, Montreal Neurological Institute; CBF, cerebral blood flow; ReHo, regional homogeneity; A-LHON, acute Leber’s hereditary optic neuropathy; C-LHON, chronic Leber’s hereditary optic neuropathy; HCs, healthy controls.

### 3.3. CBF and ReHo changes in LHON patients

To explore the separate contributions of CBF and ReHo on the neurovascular coupling abruptions in LHON, we also compared intergroup differences in CBF and ReHo values of these regions. Both A-LHON and C-LHON showed lower CBF than the HCs in the B_CAL, while only C-LHON demonstrated lower CBF than the HCs in the L_SMC and L_MTG (*p* < 1.85e^–3^, Bonferroni correction). C-LHON showed lower CBF than A-LHON in L_SMC (*p* < 1.85e^–3^, Bonferroni correction) and L_MTG (*p* < 0.05, uncorrected), and a weak higher CBF than the A-LHON in B_CAL (*p* < 0.05, uncorrected) (Figure 2B). Moreover, C-LHON showed a higher ReHo than the HCs and A-LHON in L_MTG (*p* < 1.85e^–3^, Bonferroni correction), and a weak higher ReHo than the A-LHON in the B_CAL and L_SMC (*p* < 0.05, uncorrected). There were no differences in ReHo value between the A-LHON and HCs (*p* > 0.05) (Figure 2C).

### 3.4. Correlation between ReHo/CBF and neurovascular coupling in A-LHON and C-LHON patients

To quantify which measure (ReHo or CBF) contributes primarily to the neurovascular coupling strength, Pearson’s correlation analysis demonstrated that ReHo/CBF ratio is significantly negatively correlated with CBF in all identified brain regions in both the acute and chronic LHON (*p* < 4.17e^–3^, Bonferroni corrected). Besides, a weak positive association between ReHo/CBF ratio and ReHo was identified in only the L_SMC and L_MTG in C-LHON patients (*p* < 0.05, uncorrected) (Table 3).

**TABLE 3:** Correlation between ReHo, CBF, and ReHo/CBF ratio in A-LHON and C-LHON patients.

Group	Pearson’s correlation		Brain regions		
			B_CAL	L_SMC	L_MTG
A-LHON	ReHo/CBF ratio-CBF	*r*	–0.86	–0.77	–0.88
		*p*	8.84e^–7^**	7.30e^–5^**	2.33e^–7^**
	ReHo/CBF ratio-ReHo	*r*	–0.17	0.04	0.28
		*p*	0.47	0.87	0.23
C-LHON	ReHo/CBF ratio-CBF	*r*	–0.88	–0.89	–0.86
		*p*	1.16e-^10^**	6.50e-^11^**	1.72e-^9^**
	ReHo/CBF ratio-ReHo	*r*	0.35	0.45	0.49
		*p*	6.40e^–2^	1.23e^–2^*	5.71e^–3^*

**Multiple comparisons were corrected by a Bonferroni method with a corrected threshold of *p* < 0.05/3 ROIs/2 groups/2 metric pairs = 4.17e^–3^; *nominal *p* < 0.05. B_CAL, bilateral calcarine fissure and surrounding cortex; L_MTG, left middle temporal gyrus; L_SMC, left sensorimotor cortex; CBF, cerebral blood flow; ReHo, regional homogeneity; A-LHON, acute Leber’s hereditary optic neuropathy; C-LHON, chronic Leber’s hereditary optic neuropathy.

### 3.5. Correlation between neurovascular coupling and clinical variables

**FIGURE 3:**
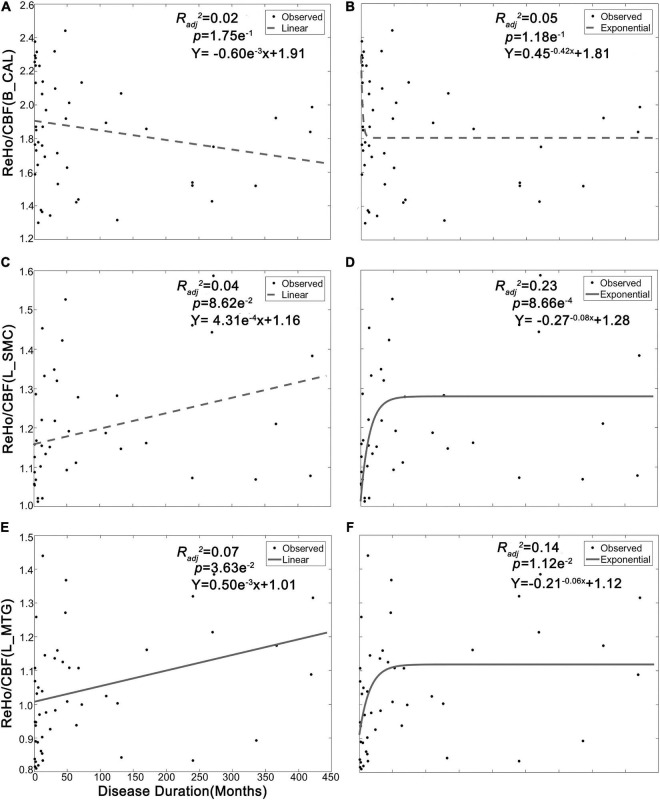
Correlation between neurovascular coupling strength and disease duration of LHON. CBF, cerebral blood flow; ReHo, regional homogeneity; A-LHON, acute Leber’s hereditary optic neuropathy; C-LHON, chronic Leber’s hereditary optic neuropathy; B_CAL, bilateral calcarine fissure and surrounding cortex; L_MTG, left middle temporal gyrus; L_SMC, left sensorimotor cortex.

A significantly positive exponential correlation was identified between the ReHo/CBF ratio of L_SMC and disease duration (*Radj*^2^ = 0.23, *p* = 8.66e^–4^, Bonferroni correction) (Table 4 and Figure 3). A weak positive exponential correlation was also identified between the ReHo/CBF ratio of L_MTG and disease duration (*Radj*^2^ = 0.14, *p* = 1.12e^–2^, uncorrected). There was no association between the ReHo/CBF ratio of B_CAL and disease duration in either the linear or non-linear model (*p* > 0.05, Bonferroni correction) (Table 4). There was no statistical correlation between ReHo/CBF ratio and neuro-ophthalmological metrics (*p* > 0.05, Bonferroni correction), though some uncorrected nominal significant findings were identified (Table 4).

**TABLE 4:** Correlation between ReHo/CBF ratio and clinical measures.

X	Model		B_CAL	L_SMC	L_MTG
Disease duration	Linear	*Radj* ^2^	0.02	0.04	0.07
		*p*	1.75e^–1^	8.62e^–2^	3.63e^–2^*
		Fit Eq.	*Y* = -0.60e^–3^x + 1.91	*Y* = 4.31e^–4^x + 1.16	*Y* = 0.50e^–3^x + 1.01
	Exponential	*Radj* ^2^	0.05	0.23	0.14
		*p*	1.18e^–1^	8.66e^–4^**	1.12e^–2^*
		Fit Eq.	*Y* = 0.45^–0.42^x + 1.81	*Y* = -0.27^–0.08^x + 1.28	*Y* = -0.21^–0.06^x + 1.12
MS	Linear	*Radj* ^2^	0.10	–3.12e^–3^	–0.01
		*p*	2.98e^–2^*	3.51e^–1^	4.75e^–1^
		Fit Eq.	*Y* = -0.02x + 2.04	*Y* = -3.03e^–3^x + 1.26	*Y* = -2.17e^–3^x + 1.09
	Exponential	*Radj* ^2^	0.12	–0.02	–0.02
		*p*	3.02e^–2^*	5.91e^–1^	5.28e^–1^
		Fit Eq.	*Y* = 0.574e^–0.22^x + 1.75	*Y* = 0.11e^–0.09^x + 1.17	*Y* = 0.13e^0.20^x + 1.03
MD	Linear	*Radj* ^2^	0.11	3.94e^–4^	–2.16e^–3^
		*p*	1.53e^–2^*	3.18e^–1^	3.43e^–1^
		Fit Eq.	*Y* = 0.01x + 1.64	*Y* = 3.46e^–3^x + 1.15	*Y* = 3.41e^–3^x + 1.00
	Exponential	*Radj* ^2^	0.15	–0.02	–0.03
		*p*	1.02e^–2^*	6.01e^–1^	6.44e^–1^
		Fit Eq.	*Y* = 6.09e^–3^e^0.15^x + 1.74	*Y* = -0.15e^0.04^x + 1.29	*Y* = -4.58e^6.13e–4^x + 5.59
LV	Linear	*Radj* ^2^	–0.02	0.02	–0.01
		*p*	7.23e^–1^	1.87e^–1^	5.90e^–1^
		Fit Eq.	*Y* = 6.98e^–4^x + 1.85	*Y* = 2.37e^–3^x + 1.16	*Y* = 6.43e^–4^x + 1.04
	Exponential	*Radj* ^2^	–0.04	0.19	0.16
		*p*	7.85e^–1^	5.09e^–3^*	1.03e^–2^*
		Fit Eq.	*Y* = -0.14e^–0.07^x + 1.91	*Y* = -0.52e^–0.19^x + 1.26	*Y* = -0.49e^0.21^x + 1.10
RNFL thickness	Linear	*Radj* ^2^	–0.02	0.10	0.09
		*p*	8.79e^–1^	1.92e^–2^*	2.51e^–2^*
		Fit Eq.	*Y* = 0.30e^–3^x + 1.86	*Y* = -1.96e^–3^x + 1.39	*Y* = -2.21e^–3^x + 1.23
	Exponential	*Radj* ^2^	–0.05	0.18	0.07
		*p*	9.88e^–1^	5.42e^–2^	8.24e^–2^
		Fit Eq.	*Y* = 0.86e^4.41e–4^x + 0.10	*Y* = 0.70e^–0.02^x + 1.07	*Y* = -0.24e^0.01^x + 1.43

**Multiple comparisons were corrected by a Bonferroni method with a corrected threshold of *p* < 0.05/3 ROIs/5 clinical measures/2 regression models = 1.67e^–3^; *nominal *p* < 0.05. CBF, cerebral blood flow; ReHo, regional homogeneity; A-LHON, acute Leber’s hereditary optic neuropathy; C-LHON, chronic Leber’s hereditary optic neuropathy; HCs, healthy controls; MD, mean defect; MS, mean sensitivity; LV, loss of variance; RNFL, retinal nerve fiber layer; B_CAL, bilateral calcarine fissure and surrounding cortex; L_MTG, left middle temporal gyrus; L_SMC, left sensorimotor cortex.

## 4. Discussion

To our knowledge, this is the first study that reported the neurovascular coupling abnormality in LHON. We found that both the acute and chronic LHON patients had abnormally higher neurovascular coupling strength in the primary visual cortex, which was accompanied by reduced CBF while preserved ReHo. Besides, only chronic LHON showed an abnormally higher neurovascular coupling strength in the sensorimotor and auditory areas with a dramatically reduced CBF and weak increased ReHo. Finally, we found that neurovascular coupling in the sensorimotor cortex was exponentially correlated with disease duration. These findings suggested that brain neurovascular “dyscoupling” within and outside the visual system may be an important neurological mechanism for LHON.

The primary visual cortex is one of the major involved areas not only for LHON but also for other patients with visual impairment. Previous structural studies have shown that visual impairment (including LHON) could induce secondary gray matter (Barcella et al., 2010; Qin et al., 2013; Tian et al., 2022) and white matter (Milesi et al., 2012; Wang et al., 2013; Manners et al., 2015; Wang et al., 2021) impairment in the primary visual cortex. In contrast to the structural impairment, we found an increased neurovascular coupling strength in the primary visual cortex. Furthermore, the increased neurovascular coupling was accompanied by decreased CBF and preserved intrinsic neuronal activity and had a negative correlation between CBF and neurovascular coupling in C-LHON and A-LHON; moreover, a weak correlation was identified between neurovascular coupling and ReHo in only the non-visual areas in chronic LHON, indicating the changed neurovascular coupling was primarily caused by a reduced blood supply. LHON’s decreased CBF in the primary visual cortex was consistent with early studies showing reduced glucose metabolism in this area of late-onset blindness (Veraart et al., 1990). The increased neurovascular coupling in the primary visual cortex indicated that its spontaneous neuronal activity per unit of CBF was more efficient in LHON than in the sighted controls.

Combined with the findings that LHON patients had no significant changes in regional intrinsic neuronal activity in the primary visual cortex, we speculated that the increased neurovascular coupling might reflect the compensatory plasticity in response to visual loss. This hypothesis was supported by early studies showing strengthened functional connectivity in the atrophied primary visual cortex in chronic LHON (Rocca et al., 2011; Tian et al., 2022) and in other visual-deprived people (Qin et al., 2015; Ma et al., 2016; Liu et al., 2017). Moreover, early studies reported that the primary visual cortex of visual-deprived subjects participated in processing non-visual information, such as auditory information (Arno et al., 2001; Poirier et al., 2006; Voss et al., 2008; Renier et al., 2010; Collignon et al., 2011), somatosensory information (Wittenberg et al., 2004; Sadato, 2005; Kupers et al., 2011), and more complex cognitive activities (Burton et al., 2002; Sadato et al., 2002; Amedi et al., 2003), suggesting that the atrophied primary visual cortex after visual impairment also preserve functions flexible to other sensory modalities. In summary, our findings provide a potential explanation for the compensatory plasticity of the primary visual cortex in response to visual loss: on the one hand, some neurons undergo secondary degeneration caused by the deafferentation of visual inputs; on the other side, the remaining occipital neurons undergo compensatory plasticity to process information *via* other modalities by strengthening the neurovascular coupling to improve the processing efficiency of these spared neurons.

Interestingly, the strengthened neurovascular coupling was also identified in the non-visual sensory areas (such as the sensorimotor and auditory systems) but only in chronic LHON patients. Besides, the strengthened neurovascular coupling in these non-visual sensory areas was accompanied by dramatically reduced CBF and a weak enhanced spontaneous neuronal activity, which was also confirmed by the negative correlation between CBF and neurovascular coupling and the positive correlation between ReHo and neurovascular coupling in non-visual areas of C-LHON. Early studies had reported blind people demonstrated strengthened tactile and auditory perception (D’Angiulli and Waraich, 2002; Fieger et al., 2006; Collignon et al., 2009), non-visual task-evoked activity in the temporal and sensorimotor cortex (Burton et al., 2003), and corticospinal tract integrity (Yu et al., 2007; Wang et al., 2013). Similar findings were also shown in LHON patients. For example, one recent study reported the betweenness centrality of the left precentral gyrus in LHON became more important, suggesting that the sensorimotor area became more important as a network hub (Jonak et al., 2021). Besides, Rocca et al. (2011) reported enhanced functional connectivity in the auditory network and a higher number of clusters in the right auditory cortex in LHON patients. Thus, the strengthened neurovascular coupling of these non-visual sensory areas might explain the experience-dependent plasticity of these regions in more efficiently processing non-visual signals, as LHON patients have to rely on more somatosensory/auditory inputs to access the outside world.

On the association between strengthened neurovascular coupling and disease duration, we found that the visual and non-visual areas exhibit completely different patterns: the non-visual areas (both sensorimotor cortex and associated auditory areas) demonstrated a positively exponential correlation between the neurovascular coupling and disease duration, but the primary visual areas showed no correlation. One possible explanation for these dissociation patterns is the different plasticity underpins the deprived and spared sensory areas. For the deprived visual cortex, we speculated a “suppression unmasking” theory might predominantly drive the plasticity of this area after LHON (Pascual-Leone et al., 2005; Lewis et al., 2010; Qin et al., 2015), which hypothesized that visual deprivation “unmasks” the existing suppressed synaptic connections between the occipital and other sensory and higher-tier areas. This theory was supported by many studies showing a rapid shift of the visual areas in processing non-visual signals (rapid cross-modal plasticity) after several days of blindfolding (Merabet et al., 2008; Radziun et al., 2022). The unmasking-driven rapid cross-modal plasticity can explain why we did not observe an association between enhanced neurovascular coupling and disease duration.

In contrast, for the spared non-visual sensory areas, their basic functions (i.e., tactile perception for SMC) are formed by long-term development and stay relatively stable after maturity. Therefore, the potential of experience-driven plasticity of these matured cortices is limited and slow (Desai et al., 2002; Kral, 2013), and should be stimulated by stronger and longer inputs after LHON, just like what happens in long-term blind people (D’Angiulli and Waraich, 2002; Fieger et al., 2006; Collignon et al., 2009), tax drivers (Maguire et al., 2000; Wang et al., 2015), musicians (Elmer et al., 2013), and opera experts (Zhao et al., 2020). However, we could not exclude the possibility of other compensatory mechanisms (i.e., unmasking) of these regions in response to direct damage to the non-visual systems, as some scholars have reported that LHON patients also suffer auditory and sensorimotor dysfunctions, such as hearing impairment (Ceranic and Luxon, 2004; Rance et al., 2012; Leng et al., 2015), myoclonic epilepsy (La Morgia et al., 2008), dystonia (Saracchi et al., 2013), cerebellar ataxia (Funakawa et al., 1995), and psychomotor regression (Grazina et al., 2007), etc.

Some limitations should be mentioned. First, to control for the inaccurate absolute CBF quantification caused by the variabilities in labeling efficiency using single labeling delay time across subjects and voxels, we used a relative CBF by dividing by the brain global mean of that subject. Although this strategy is commonly used in the voxel-wise analysis in early CBF studies (Zhu et al., 2015; Zou et al., 2015), it is preferable to use a precise absolute CBF based on multiple labeling strategies to measure the dose relationships between CBF and neuronal activity. Second, although we observed neurovascular coupling differences between acute and chronic LHON, a longitudinal design is preferable to sketch the dynamic evolution of neurovascular coupling of this disease.

In summary, LHON patients demonstrated abnormally higher brain neurovascular coupling in both the visual area and non-visual sensory areas, indicating increased CBF utilization rates in these areas for spontaneous neuronal activity in LHON.

## Data availability statement

The original contributions presented in this study are included in the article/Supplementary material, further inquiries can be directed to the corresponding authors.

## Ethics statement

The studies involving human participants were reviewed and approved by the Ethics Committees of Henan Provincial People’s Hospital. Written informed consent to participate in this study was provided by the participants’ legal guardian/next of kin.

## Author contributions

WQ, CY, and DS: study design. LW, QT, DS, and KF: data gathering. YJ, HD, and WQ: data analysis. YJ, WQ, and CY: drafting and revision. All authors contributed to the article and approved the submitted version.

## Footnotes

1
http://www.fil.ion.ucl.ac.uk/spm

2
http://rfmri.org/DPABI

3
https://www.fil.ion.ucl.ac.uk/spm/software/download

4
https://www.ibm.com/analytics/spss-statistics-software
