# Supplementary material for: Aberrant neurovascular coupling in Leber’s hereditary optic neuropathy: Evidence from a multi-model MRI analysis
Source: Front Neurosci. 2023 Jan 10;16:1050772. doi: 10.3389/fnins.2022.1050772 (PMC9871937; doi:10.3389/fnins.2022.1050772)
Supplement: Supplementary file 1 [file Data_Sheet_1.pdf]

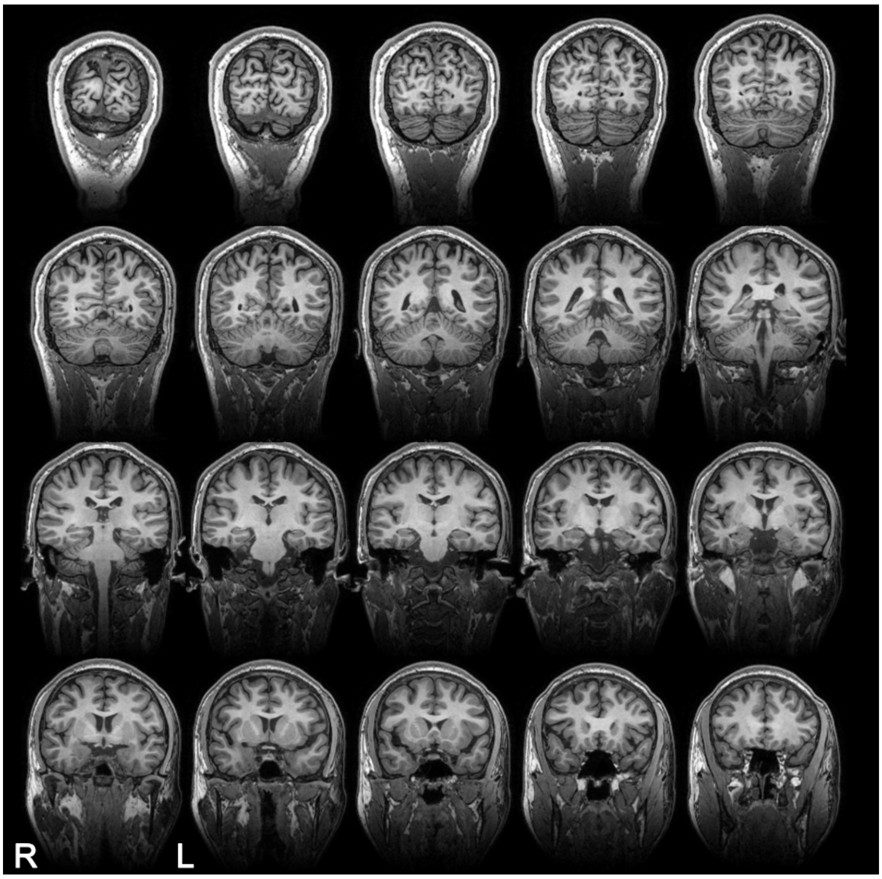

T1W coronal of brain

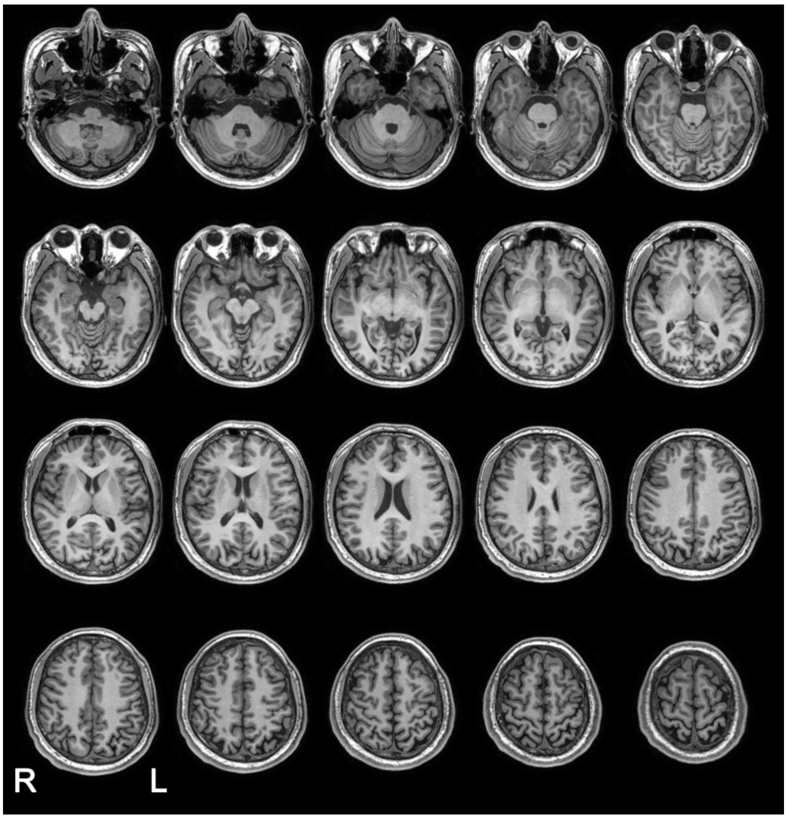

T1W transversal of brain

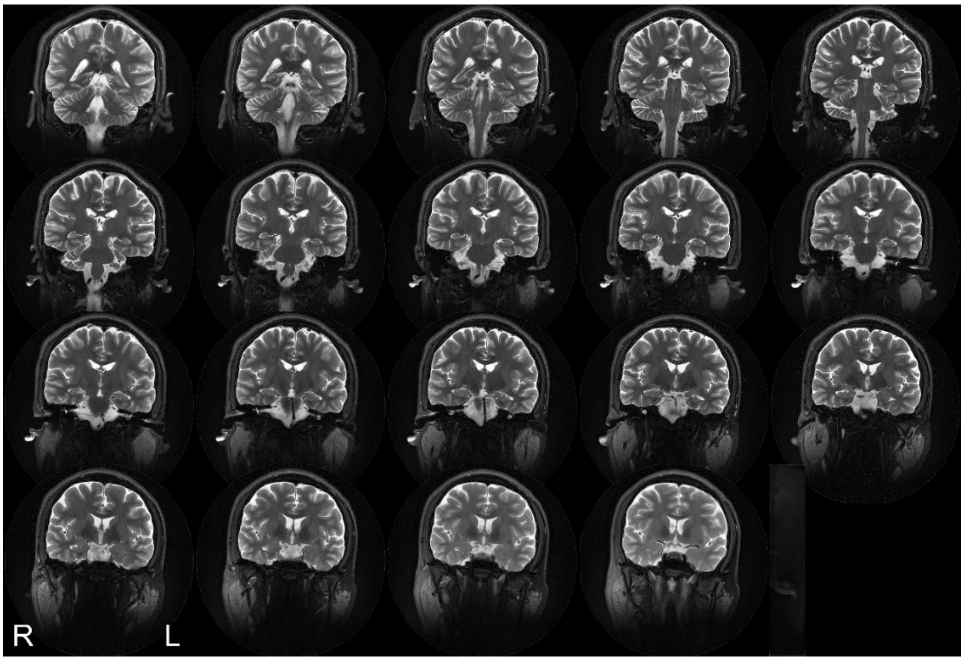

T2W coronal of brain

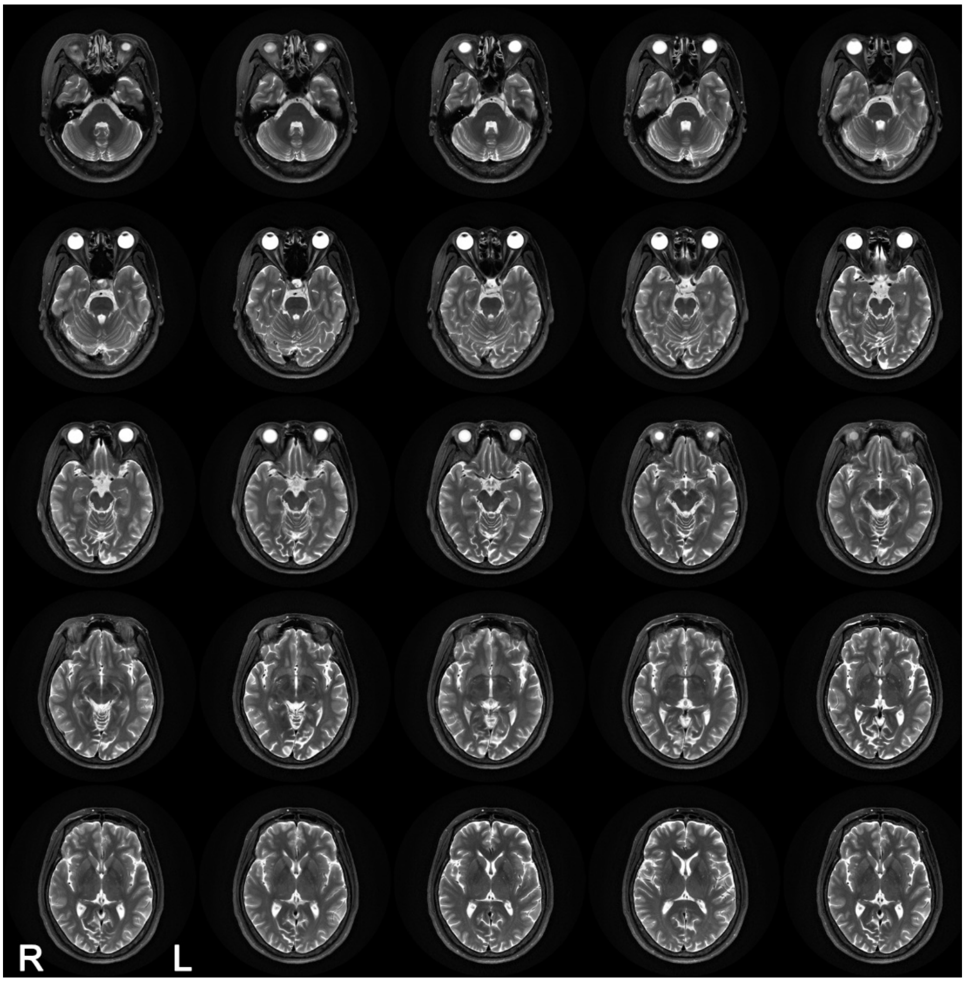

T2W transversal of brain

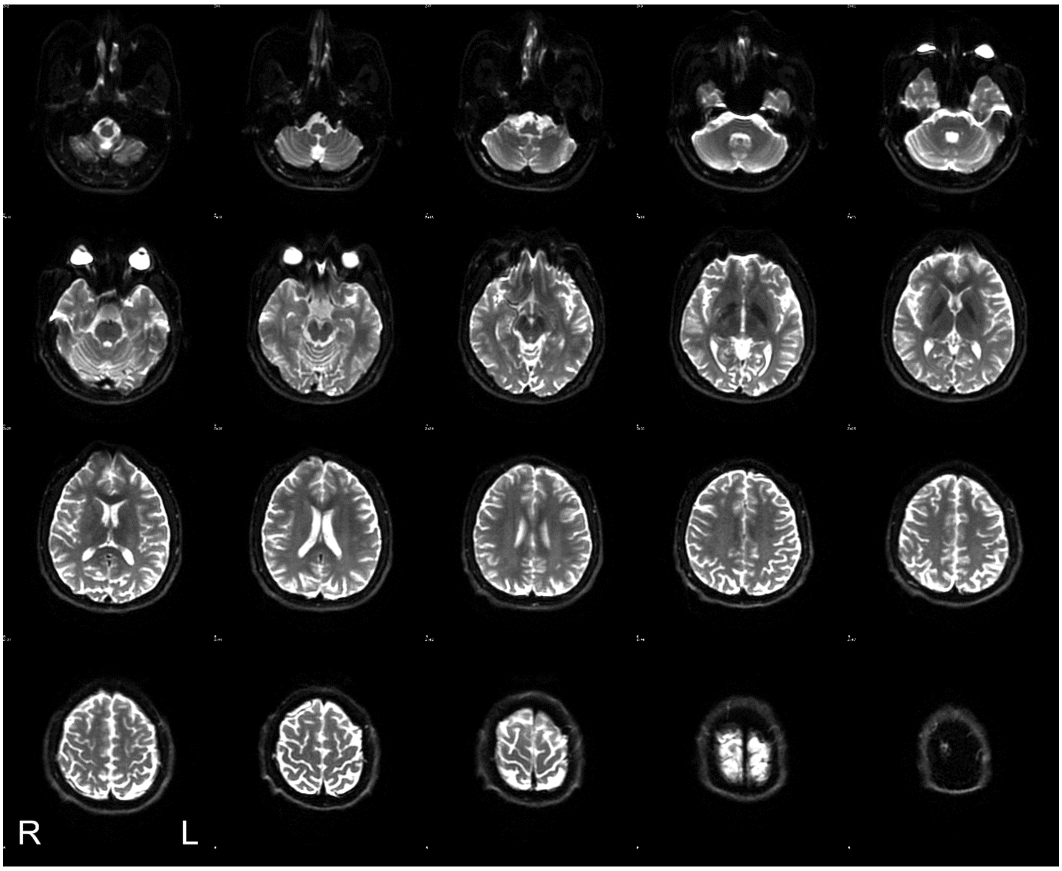

DTI transversal of brain

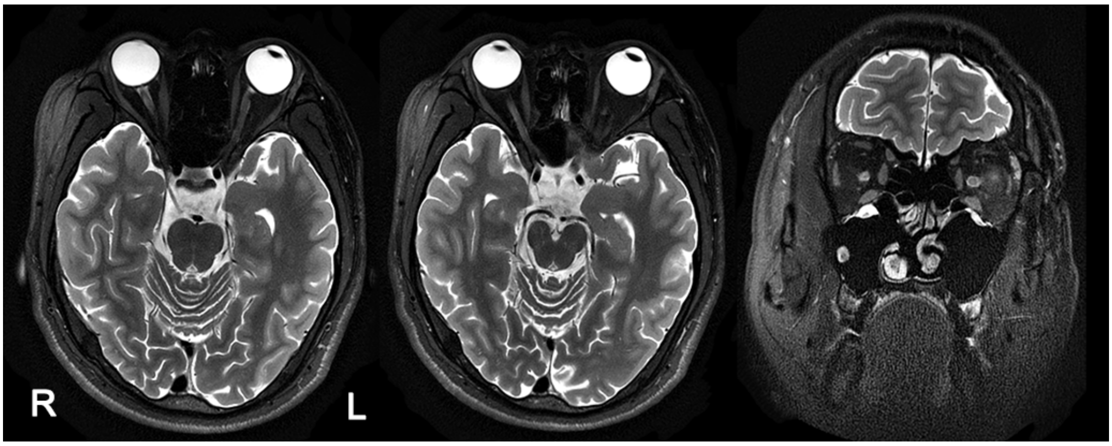

Fat saturation T2W transversal of optical nerves

Supplementary Figure1. T1, T2, DTI, optic and acoustic nerve tomography of an A-LHON patient. Note: Male, 19 years old, m.11778G > A. Disease duration of the left eye was 3 weeks, and the disease duration of the right eye was 3 months. The average thickness of the left RNFL was 142μm, and the right RNFL was 101μm.

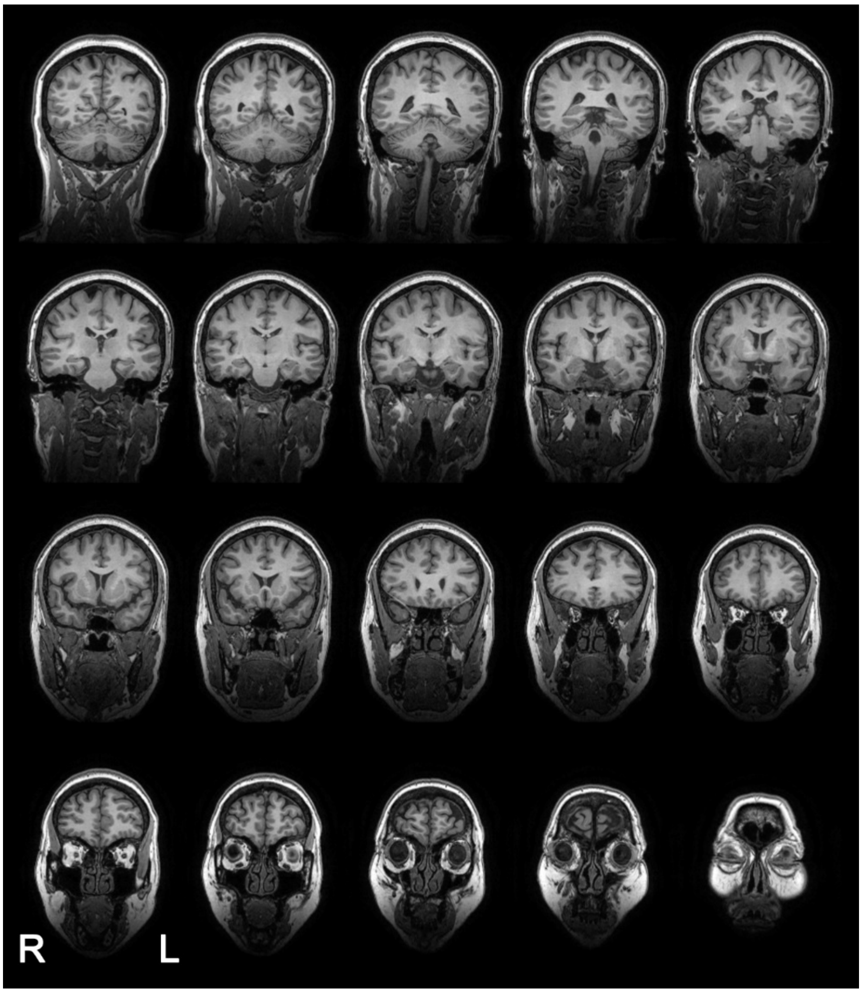

T1W coronal of brain

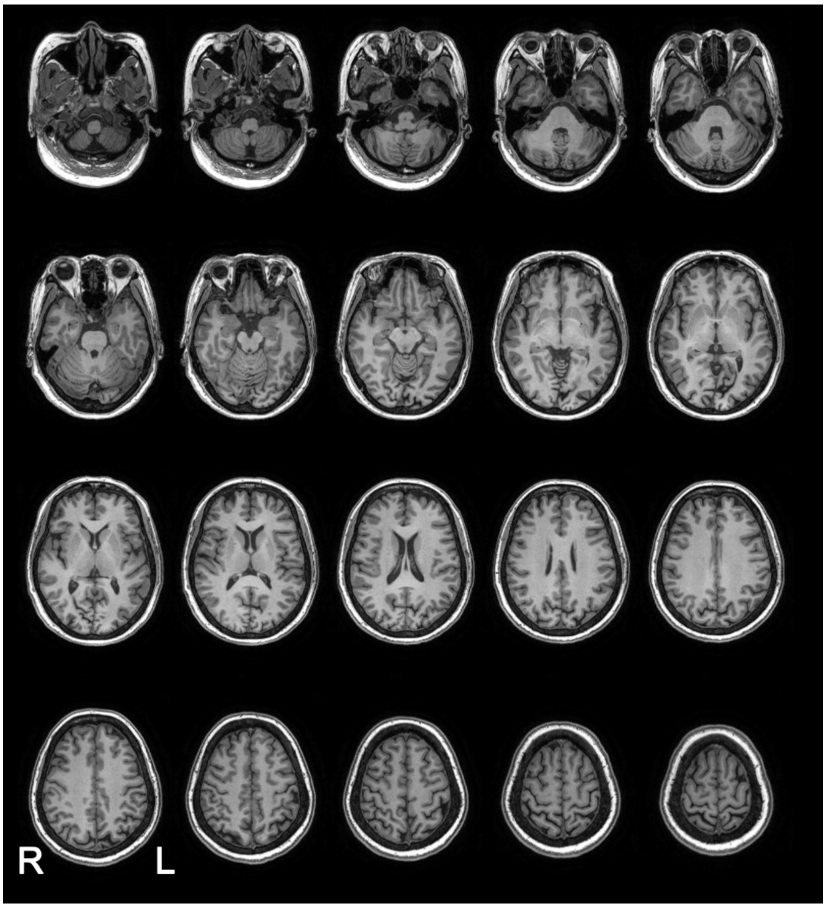

T1W transversal of brain

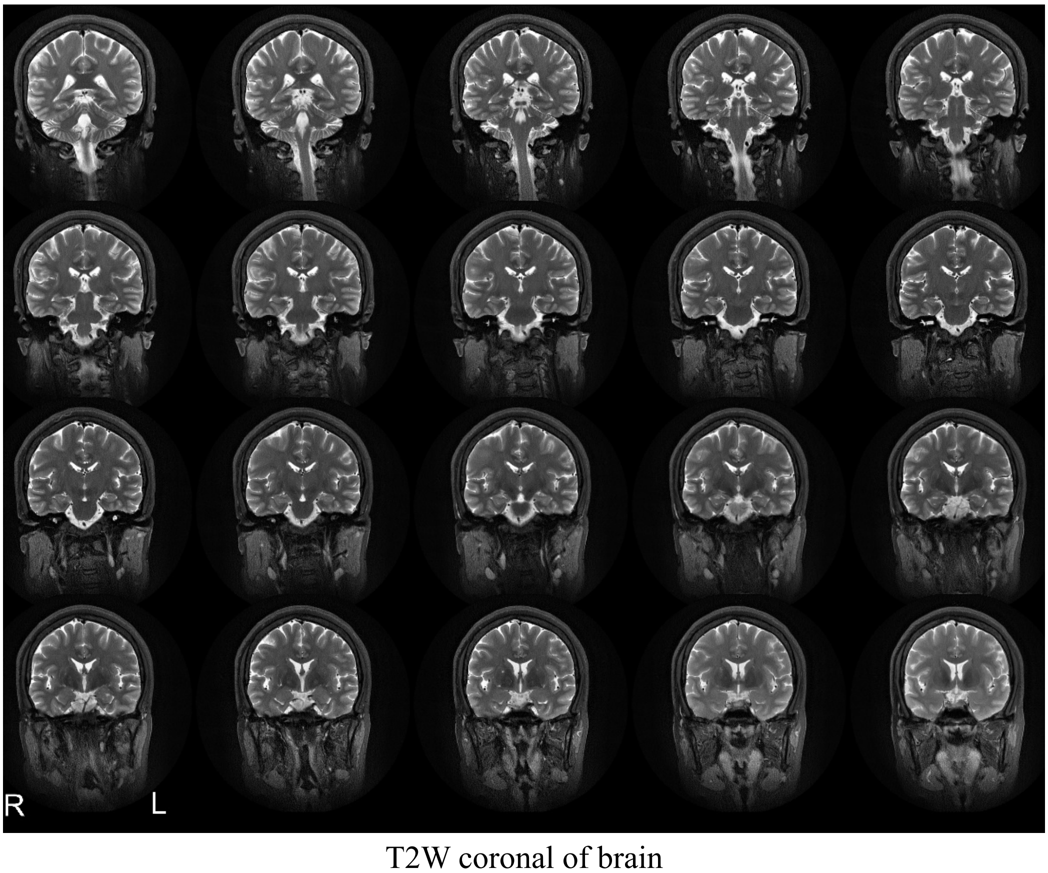

T2W coronal of brain

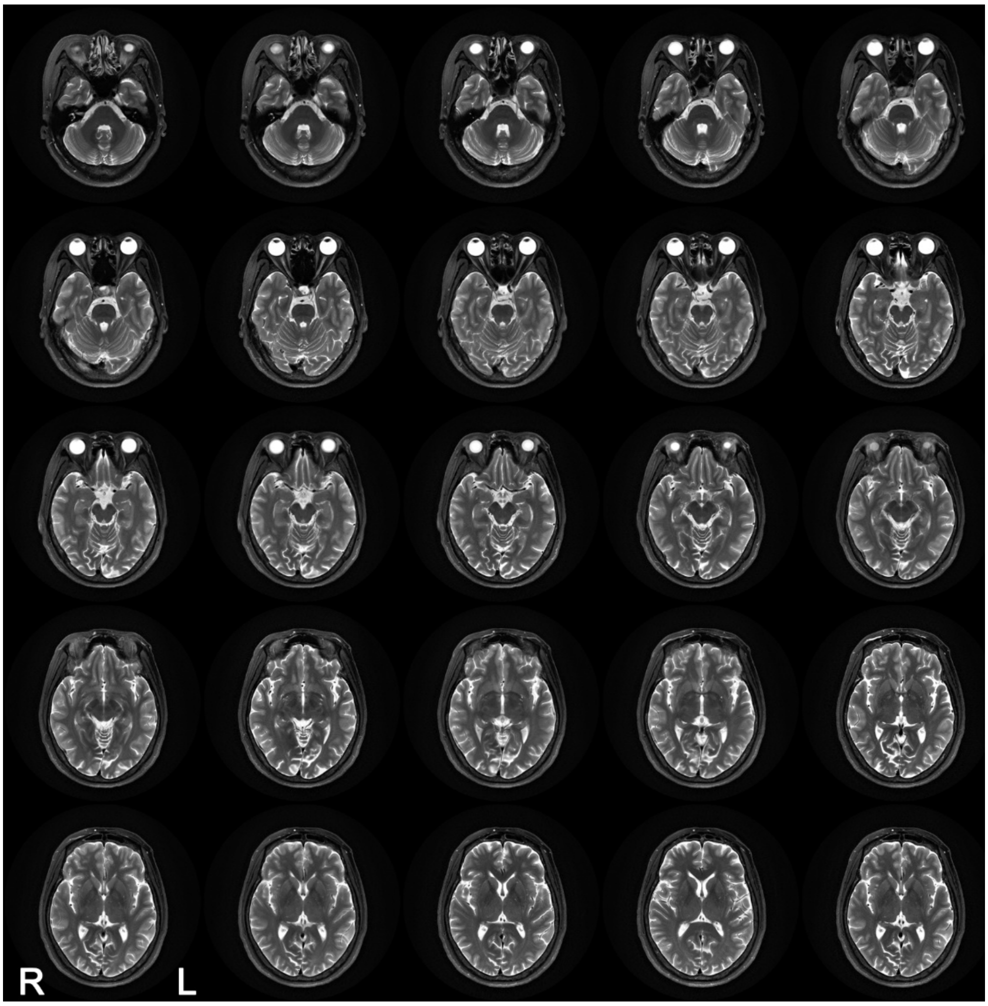

T2W transversal of brain

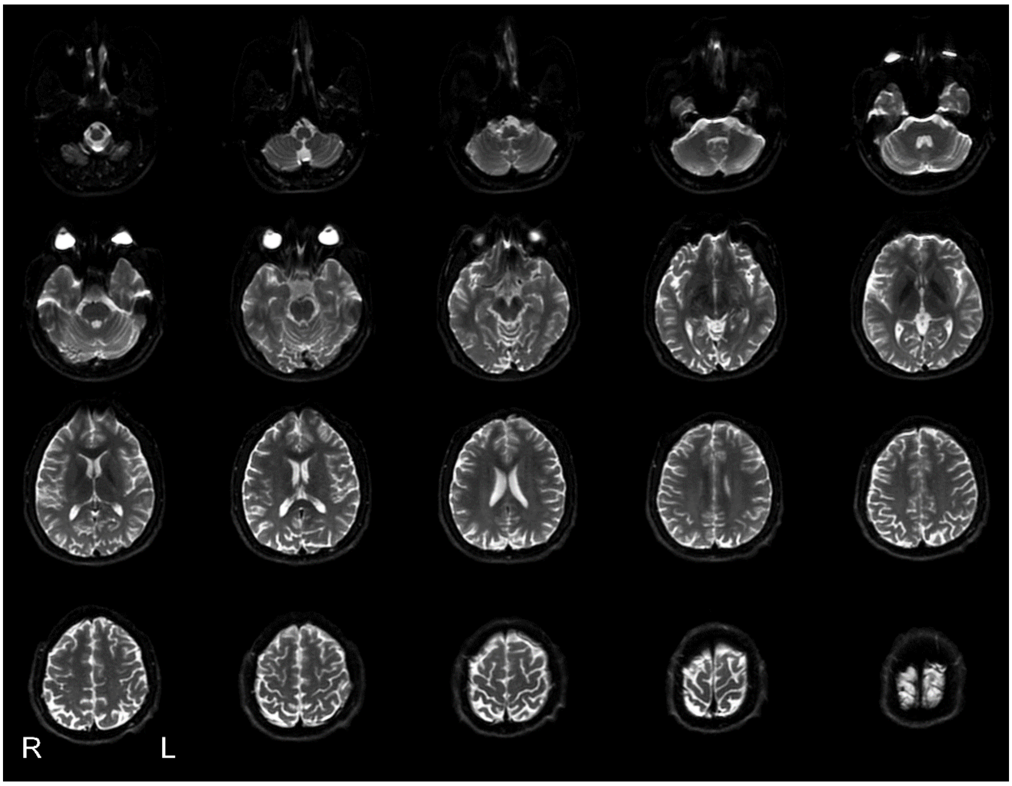

DTI transversal of brain

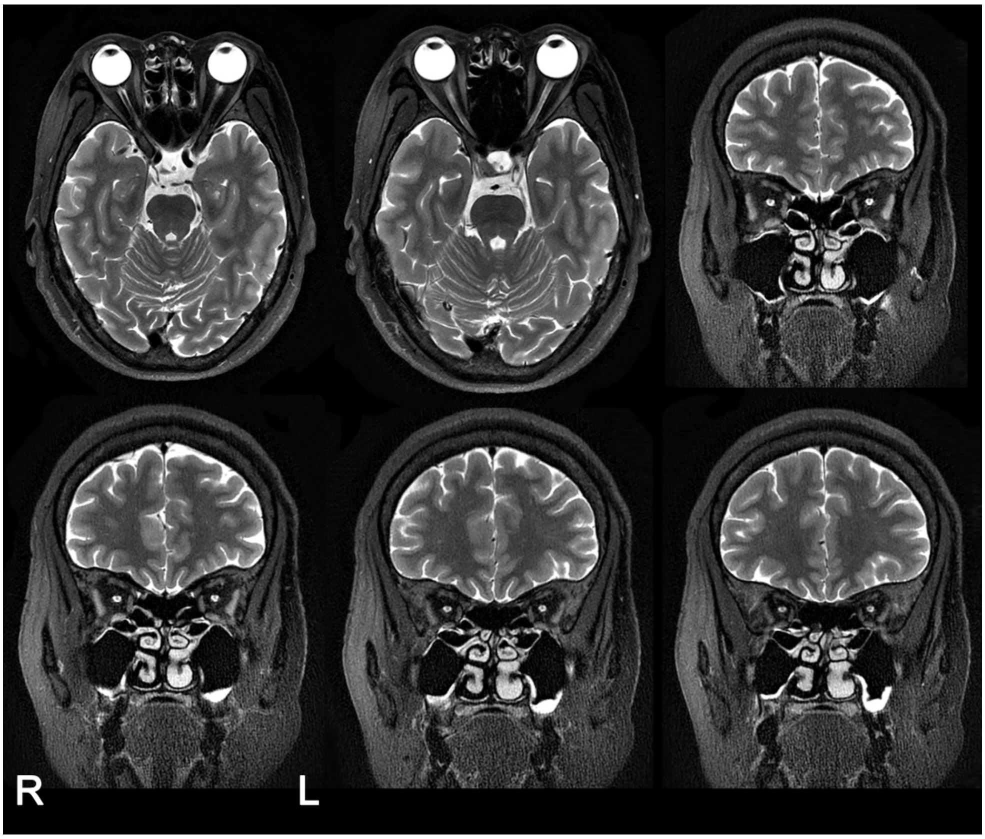

Fat saturation T2W transversal of optical nerves

Supplementary Figure2. T1, T2, DTI, optic and acoustic nerve tomography of an C-LHON patient.Note: Female, 53 years old, m.11778G > A. Disease duration of the left eye was 422 months, and the disease duration of the right eye was 442 months. The average thickness of the left RNFL was 57 μm, and the right RNFL was 58 μm.
